# Astronomy New Talent collection

**DOI:** 10.1098/rsos.221208

**Published:** 2022-10-12

**Authors:** R. J. Ivison

**Affiliations:** European Southern Observatory, Garching, Germany

We introduce the Astronomy New Talent collection, a collection of invited papers by tomorrow's leaders in astronomy and astrophysics. This collection showcases the strength of some of the research funded by the Royal Society. Comprising both research articles and review papers, this collection is a continuation of the series of commissioned New Talent collections which started with the Chemistry New Talent collection [1], which had its genesis in the unique collaboration between the Royal Society of Chemistry and Royal Society Open Science [2], and joins the Life Sciences New Talent collection [3].

The authors published within this collection are recent awardees of a Royal Society University Research Fellowship (URF) [4], Newton International Fellowship [5] or Dorothy Hodgkin Fellowship [6], and are rising stars in their areas of research. Awarded via a competitive grant application process, these grants allow outstanding future leaders in the field to conduct important research, and answer and explore significant questions within astronomy and astrophysics.

We hope that readers of the journal will explore this collection and enjoy reading these pieces as much as we did. Royal Society URF Grant Kennedy, at the University of Warwick, investigates the origin and frequency of warm dust in the so-called ‘habitable-zones’ around stars [7]. Katherine Joy, Royal Society URF at the University of Manchester, and colleagues study the timing of geological events in the lunar highlands recorded in shocked zircon-bearing clasts from Apollo 16 [8]. Newton International Fellowship holder Bingkun Yu at the University of Science and Technology of China and colleagues investigate a large dataset from the FORMOSAT-3/COSMIC satellite mission, and combine the data with ground-based observations recorded by 25 monitoring stations to derive a map of critical frequencies of Es layers (foEs) with high spatial and temporal resolution [9]. Morgan Fraser, a Royal Society URF at University College Dublin, presents a review piece studying interacting supernovae and transients that have been observed to date, and classifies and groups them together in a phenomenological framework [10]. Richard Parker from the University of Sheffield is a Dorothy Hodgkin Fellowship holder, and presents an in-depth review describing how planets form at the same time as stars, and discusses the external effects that can be detrimental to young planetary systems [11].

We look forward to growing and expanding this collection from more contributors who are funded by the Royal Society. This collection highlights some of the exciting work from tomorrow's leaders in the field who are delving into the most pertinent questions within astronomy.

These papers are all curated at https://royalsocietypublishing.org/topic/special-collections/astronomy-new-talent [12].

## References

[1] Royal Society Open Science. 2022 Chemistry ‘New Talent’. See https://royalsocietypublishing.org/topic/special-collections/new-talent (accessed 22 August 2022). 10.1098/rsos.251023 PMC12212995 40607181

[2] Royal Society Open Science. 2022 Chemistry. See https://royalsocietypublishing.org/rsos/chemistry (accessed 22 August 2022). 10.1098/rsos.251023 PMC12212995 40607181

[3] Royal Society Open Science. 2022 Life Sciences ‘New Talent’. See https://royalsocietypublishing.org/topic/special-collections/life-sciences-new-talent (accessed 22 August 2022). 10.1098/rsos.251023 PMC12212995 40607181

[4] The Royal Society. 2022 University Research Fellowship. See https://royalsociety.org/grants-schemes-awards/grants/university-research/ (accessed 22 August 2022).

[5] The Royal Society. 2022 Newton International Fellowship. See https://royalsociety.org/grants-schemes-awards/grants/newton-international/ (accessed 22 August 2022).

[6] The Royal Society. 2022 Dorothy Hodgkin Fellowship. See https://royalsociety.org/grants-schemes-awards/grants/dorothy-hodgkin-fellowship/ (accessed 22 August 2022).

[7] Kennedy GM. 2020 The unexpected narrowness of eccentric debris rings: a sign of eccentricity during the protoplanetary disc phase. R. Soc. Open Sci. **7**, 200063. (10.1098/rsos.200063) 32742686 PMC7353968

[8] Joy KH, Snape JF, Nemchin AA, Tartèse R, Martin DM, Whitehouse MJ, Vishnyakov V, Pernet-Fisher JF, Kring DA. 2020 Timing of geological events in the lunar highlands recorded in shocked zircon-bearing clasts from Apollo 16. R. Soc. Open Sci. **7**, 200236. (10.1098/rsos.200236) 32742691 PMC7353986

[9] Yu B, Scott CJ, Xue X, Yue X, Dou X. 2020 Derivation of global ionospheric Sporadic E critical frequency (foEs) data from the amplitude variations in GPS/GNSS radio occultations. R. Soc. Open Sci. **7**, 200320. (10.1098/rsos.200320) 32874629 PMC7428263

[10] Fraser M. 2020 Supernovae and transients with circumstellar interaction. R. Soc. Open Sci. **7**, 200467. (10.1098/rsos.200467) 32874641 PMC7428271

[11] Parker RJ. 2020 The birth environment of planetary systems. R. Soc. Open Sci. **7**, 201271. (10.1098/rsos.201271) 33391806 PMC7735350

[12] Royal Society Open Science. 2022 Astronomy ‘New Talent’. See https://royalsocietypublishing.org/topic/special-collections/astronomy-new-talent (accessed 22 August 2022). 10.1098/rsos.251023 PMC12212995 40607181

